# A Comprehensive Analysis of a Social Intelligence Dataset and Response Tendencies Between Large Language Models (LLMs) and Humans

**DOI:** 10.3390/s25020477

**Published:** 2025-01-15

**Authors:** Erika Mori, Yue Qiu, Hirokatsu Kataoka, Yoshimitsu Aoki

**Affiliations:** 1 National Institute of Advanced Industrial Science and Technology (AIST), Tsukuba 305-8560, Japan; erika-mori@aist.go.jp (E.M.); qiu.yue@aist.go.jp (Y.Q.); hirokatsu.kataoka@aist.go.jp (H.K.); 2 Department of Electronics and Electrical Engineering, Faculty of Science and Technology, Keio University, 3-14-1, Hiyoshi, Kohoku-ku, Yokohama 223-8522, Japan

**Keywords:** human–robot interaction, social intelligence, understanding human behavior, emotion recognition, large language models (LLMs), VideoQA

## Abstract

In recent years, advancements in the interaction and collaboration between humans and have garnered significant attention. Social intelligence plays a crucial role in facilitating natural interactions and seamless communication between humans and Artificial Intelligence (AI). To assess AI’s ability to understand human interactions and the components necessary for such comprehension, datasets like Social-IQ have been developed. However, these datasets often rely on a simplistic question-and-answer format and lack justifications for the provided answers. Furthermore, existing methods typically produce direct answers by selecting from predefined choices without generating intermediate outputs, which hampers interpretability and reliability. To address these limitations, we conducted a comprehensive evaluation of AI methods on a video-based Question Answering (QA) benchmark focused on human interactions, leveraging additional annotations related to human responses. Our analysis highlights significant differences between human and AI response patterns and underscores critical shortcomings in current benchmarks. We anticipate that these findings will guide the creation of more advanced datasets and represent an important step toward achieving natural communication between humans and AI.

## 1. Introduction

The potential for collaboration between humans and AI has garnered increasing interest in recent years. A critical foundation for achieving natural and seamless interactions in this domain is social intelligence, a concept that has been extensively studied across disciplines such as psychology, sociology, and artificial intelligence [1,2,3,4,5,6,7]. Social intelligence plays a vital role in understanding individual and group human behaviors, interpreting non-verbal cues such as facial expressions and gestures, anticipating human needs, providing context-aware assistance, and enabling smooth collaboration with humans in diverse scenarios and activities.

In the context of AI, social intelligence is increasingly recognized as a cornerstone for advancing human–robot interaction and broader AI applications [8,9,10,11,12]. Recent developments in robotics have emphasized the importance of equipping systems with empathetic capabilities to interpret non-verbal human behaviors such as gestures and facial expressions [13,14]. The study by Lee et al. [13] proposes a new method for integrating non-verbal cues into social robots, aiming to enhance their empathetic capabilities. Additionally, Sotopia [14] provides an interactive benchmark for assessing social intelligence and evaluating the ability of agents to achieve social goals in a multidimensional manner.

Similarly, advancements in vision-based AI have enhanced emotion recognition and the analysis of social interactions, employing datasets [15,16,17] that scrutinize human behavior through images and videos. The evolution of these technologies has been accelerated through the utilization of multiple datasets, particularly in the fields of emotional recognition and social interaction analysis. For instance, the CMU-MOSEI dataset [15], which includes over 23,500 sentence utterance videos from more than 1000 speakers, is the largest dataset for multimodal emotion analysis and recognition, offering significant improvements in speaker diversity, topic range, and total video duration (over 65 h) compared to earlier datasets such as CMU-MOSI [18], ICT-MMMO [19], and YouTube [20]. The MELD dataset [16], derived from the “Friends” TV series, includes more than 1400 dialogues and over 13,000 utterances, extending the EmotionLines dataset [21] by incorporating audio and visual modalities to cover natural dialogues across multiple speakers. The MovieQA dataset [17] aims to assess narrative understanding in films through multiple-choice questions that focus on the understanding of action and context, with one correct answer and four distractors. These benchmarks are primarily aimed at evaluating simple emotion recognition and behavioral understanding. However, the Social-IQ [22] stands out by comprehensively assessing interpersonal skills through multimodal inputs such as video, image, and audio transcriptions, evaluating social intelligence through questions about the intentions behind actions, the mood of the scene, the reasons for specific emotions, and relationships among people.

Recent studies suggest that for tasks characterized by high ambiguity, such as social interactions, including detailed information about the rationale and context in videos is beneficial. For example, the JRDB-Social dataset [23] enhances the original JRDB [24] by adding annotations at three levels—individual attributes, intra-group interactions, and the context of social groups—thus facilitating a deeper understanding of human social interactions in various settings. The Visual Commonsense Reasoning (VCR) dataset [25] expands on the visual Question Answering by capturing the reasoning process behind answers, annotating not only questions and answers but also justifications.

**Figure 1:**
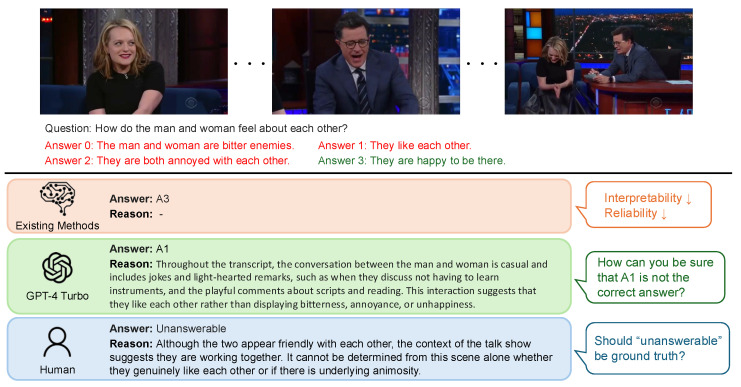
A comparison of existing methods, GPT-4 Turbo, and human responses to the Social-IQ 2.0 dataset reveals distinct differences in output characteristics. Existing methods generate only the selected options, which often lack interpretability and reliability. In contrast, GPT-4 Turbo and humans can provide justifications alongside the selected options. However, since the Social-IQ 2.0 dataset does not include ground truth labels for reasoning, it is not possible to evaluate the validity of these justifications. This article addresses this limitation by comparing human responses with those from models.

Despite the presence of numerous ambiguous and subjective QA pairs in Social-IQ [22] and its enhanced version, Social-IQ 2.0 [26], only the correct answer options are labeled, which constrains the reliability and interpretability of results (see Figure 1). Moreover, existing approaches in the Social-IQ and Social-IQ 2.0 datasets tend to rely on end-to-end models that solely depend on correct answer labels, limiting their adaptability to various scenarios and necessitating dataset-specific training [27,28,29,30,31,32].

In our study, we address the limitations of existing datasets and evaluation methods by deploying large language models (LLMs), such as GPT-4 Turbo and GPT-4o [33], in the Social-IQ 2.0 benchmark. These models, evaluated in a zero-shot setting, achieve accuracy comparable to traditional dataset-specific approaches without necessitating further training. Employing supplementary annotation data from the Social-IQ 2.0 dataset allows for analyses that extend beyond simple QA accuracy, including detailed examinations of the accuracy and response patterns of both humans and LLMs. This provides valuable insights into the potential applications of LLMs in video-based tasks and their relative effectiveness compared to human benchmarks.

In summary, the contributions of this paper are as follows:

- We demonstrated that zero-shot approaches using LLMs achieve performance comparable to state-of-the-art methods that require dataset-specific training, highlighting their robustness.
- Through detailed comparisons using supplementary annotations, we identified differences in response rationales and patterns between humans and LLMs, offering insights beyond simple QA performance metrics.
- We conducted in-depth analyses on the optimal number of frames, sampling strategies, and caption types, providing practical guidelines for integrating LLMs into video-based AI tasks.

## 2. Related Work

### 2.1. Social Intelligence in Artificial Intelligence

#### 2.1.1. Social Intelligence

Social intelligence [1,6,7] and its evaluation [2,3,4,5] have been extensively studied across disciplines such as psychology, sociology, anthropology, and cognitive science. Social intelligence encompasses a wide range of research topics, including the recognition of human emotions, attitudes, personalities, mental states, and facial expressions at the individual level, as well as the recognition of human relationships, individual and group behaviors, social situation understanding, and memory for human coreference [22,26]. Achieving a high level of social intelligence often requires continuous observation of human group activities through videos, as well as understanding the evolving dynamics of conversations, including both auditory elements like tone of voice and visual cues such as facial expressions and gestures.

#### 2.1.2. Recent Social Intelligence in Artificial Intelligence

Understanding human social behaviors is a fundamental capability for various robotic applications and the ultimate realization of general AI. This topic has been extensively studied in robotics [13,34,35,36]. Dautenhahn [34] and Bartneck et al. [35] explored the evaluation of social intelligence in the context of human–robot interaction, aiming to develop robots that are comfortable, reliable, and safe for human users. Lee et al. [13] proposed robots with empathetic capabilities by enabling them to comprehend non-verbal human behaviors, such as gestures and facial expressions.

Social intelligence has recently gained significant attention in vision AI, particularly in studies focusing on understanding aspects of social intelligence from single images or videos. Emotion recognition is one of the most widely discussed components of social intelligence in vision AI [15,16,37,38]. Various datasets have been introduced to facilitate the recognition of human emotions from images or videos. For instance, Zadeh et al. [15] proposed the CMU Multimodal Opinion Sentiment and Emotion Intensity (CMU-MOSEI) dataset, designed to simultaneously analyze language, facial expressions, and voice tones. Similarly, Poria et al. [16] developed multi-human conversation datasets aimed at recognizing emotions and interactions in group conversations.

Social interaction recognition is another critical area for understanding human group behaviors. The CHAMPAGNE [39] and JRDB-Social [23] datasets focus on capturing conversations and social interactions within videos of group activities. Commonsense reasoning also plays a vital role in social intelligence, particularly when the reasoning extends beyond the information directly available in videos. The Visual VCR dataset [25], for example, evaluates models’ abilities to apply external commonsense knowledge and make judgments during QA based on single images. This work has been extended by the VisualCOMET dataset [40], which analyzes commonsense reasoning in videos, including the causes and consequences of human behaviors.

Additionally, abductive reasoning has been explored in datasets such as the Abduction of Sherlock Holmes [41] and VisualABC [42], which focus on reasoning from images and videos, respectively. Finally, several studies aim to recognize complex human social interactions and behaviors within movie contexts [17,43,44]. These studies often require an understanding of the intricate human behaviors and interactions embedded in stories and plots, presenting a significant challenge for current AI models.

While most of the aforementioned studies focus on specific aspects of social intelligence, the SocialIQ 1.0 [22] and SocialIQ 2.0 [26] datasets aim to provide a more comprehensive evaluation. These datasets assess social intelligence by requiring models to answer questions about group human activities in input videos, addressing four key dimensions: judgments in social situations, recognition of human mental states and attributes, understanding processes of human intelligent behavior, and memory for coreference and grounding. Both datasets are formatted to evaluate the performance of models through four-choice QA and only include annotations for QA labels and correct answer pairs, leaving the rationale or justification behind the answers unclear. This can be said to evaluate the social intelligence of AI, particularly LLMs, in a limited way compared to datasets like VCR [25], which are annotated with rationales, or Sotopia [14], which incorporates human evaluations through various metrics. Therefore, we used a custom dataset that includes human responses and rationales for Social-IQ 2.0 to conduct a detailed comparison with humans and clearly identified differences in answer accuracy and response tendencies.

### 2.2. Social Intelligence Recognition Models

Several methods have been developed to comprehend social intelligence and answer questions in the Social IQ 1.0 [22] and 2.0 [26] datasets. The authors of the Multimodal Co-Attention Based Network for Question Answering (MCQA) [27] proposed a co-attention mechanism to better correlate multimodal inputs, such as video, audio, and language, present in the Social IQ datasets. However, many multimodal reasoning models tend to rely heavily on a specific modality, such as language, leading to superficial predictions. To address this, Gat et al. [28] introduced a novel regularization term to better balance multiple modalities and suppress biases. Similarly, Guo et al. [29] tackled bias in the Social IQ datasets, not through modeling but by improving the dataset itself, introducing perturbations to mitigate inherent biases.

Understanding Social IQ requires a series of reasoning abilities and inference from multiple clues, which often fluctuate during dynamic human interactions. To address this complexity, Sartzetaki et al. [30] proposed a step-by-step reasoning modular network to decompose the reasoning process and improve accuracy. The authors of Just Ask Plus [45] introduced a simple yet efficient model that leverages video transcripts to solve tasks in the Social IQ dataset, achieving state-of-the-art results in the zero-shot scenario. While most methods focus on holistic improvements to the Social IQ datasets, the authors of Face-to-Face Contrastive Learning [31] developed a contrastive-learning-based approach to specifically address speaking turn boundaries, enabling a better handling of multi-person interaction scenarios. Additionally, the authors of the Multi-Modal Temporal Correlated Network with Emotional Social Cues (MMTC-ESC) [32] achieved state-of-the-art performance by combining contrastive learning with multimodal inputs and emotional social cues.

However, most of these methods require training on the Social IQ datasets, making them relatively vulnerable to unknown cases where the input distribution—either in vision or language—differs from the original datasets. This limitation reduces their applicability to scenarios involving social intelligence tasks not covered by the datasets. In contrast, our paper evaluates several of these methods as baselines and employs a zero-shot approach, which offers greater robustness to varied input data and broader applicability to unseen scenarios.

### 2.3. LLMs and Multimodal LLMs

LLMs, such as LLaMA [46] and GPT models [47], as well as Multimodal LLMs like LLaVA [48], GPT-4v/o [33], and Gemini [49], are pretrained on large-scale language corpora to comprehend language. These models demonstrate emergent abilities in various domains, including understanding context, applying commonsense reasoning, performing chain-of-thought inference, and interpreting humor. They are also believed to possess some degree of social intelligence through their language comprehension capabilities. In this paper, we select representative LLMs and Multimodal LLMs, such as GPT-4 Turbo and GPT-4o, to examine their zero-shot social intelligence recognition abilities in detail. By evaluating their performance on the SocialIQ 2.0 dataset, we analyze their ability to provide detailed answer justifications and compare their outputs with human performance and reasoning. This extensive benchmarking provides a comprehensive understanding of their capabilities in social intelligence recognition.

## 3. Experiments

This study conducted a comprehensive evaluation of the social intelligence capabilities of LLMs using the Social-IQ 2.0 dataset [26], supplemented with additional annotation data we created (Social-IQ 2.0 Sub) (available at https://github.com/edrkr96/Social-IQ-2.0-Sub accessed on 5 January 2025). The LLMs used in this study include LLaMA 3, several versions of GPT [33], and several versions of GPT-o [33]. In Section 3.1, the dataset used in this study is described, and Section 3.2 discusses the comparison of zero-shot accuracy between existing methods and the LLMs. Section 3.3 presents an analysis of patterns in correct and incorrect responses, answer tendencies, and accuracy based on problem types, comparing humans and LLMs. Finally, Section 3.4 details an ablation study examining the impact of input data types, the number of frames, types of LLMs, captioning models, and the granularity of captions on performance.

### 3.1. Datasets

The Social-IQ 2.0 dataset [26] consists of over 1000 videos, each approximately 60 s long, with an average of 6 to 7 multiple-choice (4-option) QA pairs per video. The dataset spans multiple modalities, including video, images, dialogue, and audio. Unlike questions that involve straightforward action recognition, the questions in Social-IQ 2.0 require more complex reasoning, such as understanding the intentions behind actions or interpreting the mood of a situation. In this study, Social IQ 2.0 is utilized to examine the social intelligence of LLMs by comparing their accuracy with existing methods, as outlined in Section 3.2. However, since Social-IQ 2.0 consists of multiple-choice QAs, evaluations are limited to QA accuracy alone, without the capability for detailed analysis at the level of reasoning. This might be insufficient when considering LLM-based methods that can adapt the format based on the prompt.

The Social-IQ 2.0 Sub dataset is an auxiliary dataset that we previously created to enable detailed analysis and comparison with humans at the level of reasoning. It consists of 200 randomly sampled videos from Social-IQ 2.0, divided into training (174 videos) and validation (26 videos) sets based on the same ratio as the full Social-IQ 2.0 dataset. It has been confirmed that the transcripts provided with the Social-IQ 2.0 dataset are of relatively low quality; however, to prevent this from negatively impacting the accuracy of human responses, it should be noted that the transcripts were reacquired using the Whisper (large) model [50], which is known for its high transcription accuracy. It includes annotations for 1207 questions, capturing human responses and their reasoning. The annotation data are split into two types: responses based on the video content and responses based on the transcript only. In this study, only video-based response data are used, except in Section 3.3.2.

The specific annotation fields include video name, question ID, answerability, selected options (allowing multiple selections), reasoning, rationale (selected from pre-defined categories), and the time segment of the video referenced to answer the question. Among these, we utilize only the fields for answerability, video name, question ID, selected choices, and reasoning.

### 3.2. Comparison of LLM and Existing Methods in Accuracy

The LLaMA [51] and GPT series models [33,47] were applied to the validation set of the Social-IQ 2.0 dataset. This validation set consists of 120 samples, each with available video and annotation data. Due to downloading data from YouTube, there may be a decrease in the number of available samples compared to those at the time of publication. Furthermore, the validation was conducted under the original conditions of the Social-IQ 2.0 setup, which requires selecting one answer from four choices. For each LLM, the input data included five frames sampled at equal intervals from the middle portion of the video, specifically frames 20 to 160 out of approximately 180 frames in each video. Additionally, the dataset-provided transcript files, generated by YouTube’s automatic transcription feature during download, were used as video-related information. Since LLMs other than GPT-4o and GPT-4o mini cannot directly process images, captions generated by BLIP-2 [52] were provided as inputs instead of frames.

To compare with existing methods, we used Just Ask Plus [45] as a non-trained method and DeSIQ [29] and MMTC-ESC [32] as trained methods. The results are shown in Table 1.

From Table 1 it can be inferred that the performance of LLMs is proportional to their QA accuracy on the Social-IQ benchmark. Furthermore, despite not being trained on the Social-IQ 2.0 dataset, GPT-4 Turbo and GPT-4o have achieved accuracies comparable to existing trained methods. This underscores the already substantial capability of LLM-based approaches in understanding interpersonal communication at a high level. Additionally, these results demonstrate the strong zero-shot performance of LLMs, suggesting their potential effectiveness on multimodal data related to social intelligence beyond Social-IQ 2.0. This highlights the potential importance of considering LLM-based methods when designing future datasets.

### 3.3. Comparative Analysis of LLM and Human Responses

This section provides a detailed analysis of the patterns of correct and incorrect responses, as well as answer tendencies, for humans and LLMs using the Social-IQ Sub dataset. Section 3.3.1 examines the response patterns for humans compared to GPT-4 Turbo and GPT-4o, highlighting the tendencies in their answers and the unique characteristics of the QA tasks in Social-IQ 2.0. In Section 3.3.2, we categorize question types using GPT-4 Turbo and conduct evaluations based on these classifications. Finally, in Section 3.3.3, we utilize GPT-4 Turbo to assign scores to questions based on the distinguishability of the options. The accuracy of responses is then analyzed based on the score.

#### 3.3.1. Analysis of Result Patterns

Quantitative Results for GPT-4 Turbo

Using GPT-4 Turbo, we conducted QA on the 200 videos included in the Social-IQ 2.0 Sub dataset and collected its responses and reasoning. For the video input information, we used a similar approach to Section 3.2, incorporating five sampled frames from the video and dialogue information (transcript). However, the settings for generating responses differ significantly from those in Section 3.2. To address scenarios involving insufficient input information or highly ambiguous questions, we introduced a “unanswerable” option where no choice is selected. At the same time, GPT-4 Turbo was allowed to output multiple choices. When GPT-4 Turbo determined a question to be “unanswerable”, the result was categorized as “unanswerable” rather than as a wrong answer. If multiple choices were output and the correct choice was among them, the response was considered correct.

**Figure 2:**
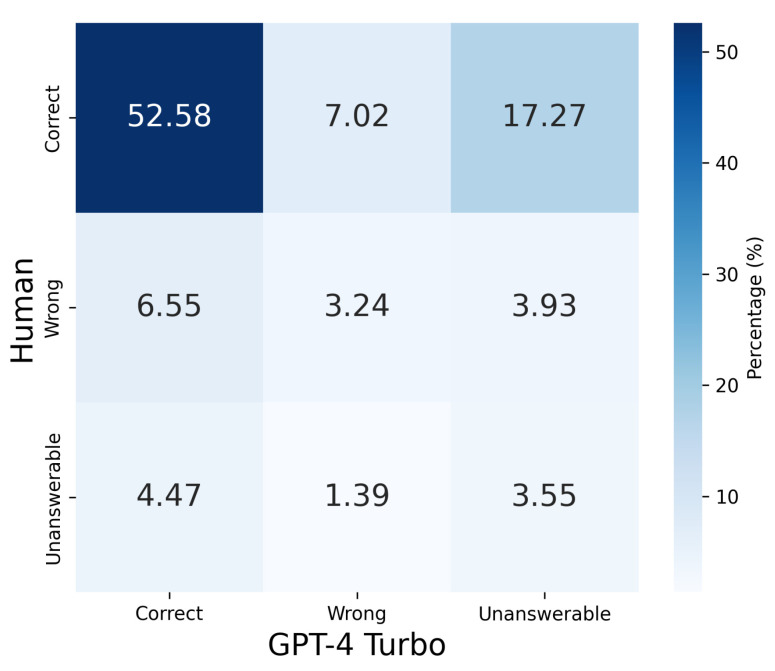
Visualization of human and GPT-4 Turbo performance. The chart illustrates the proportion of correct, wrong, and unanswerable responses in the overall dataset, with darker shades indicating higher proportions.

Using this setup, we analyzed the GPT-4 Turbo responses alongside the human response data included in the Social-IQ 2.0 Sub dataset. The analysis was conducted across nine patterns based on the three possible outcomes (correct, incorrect, unanswerable) for both humans and GPT-4 Turbo. The results are presented in Table 2 and Figure 2.

From Figure 2 and Table 2, it is evident that the response tendencies of humans and GPT-4 Turbo do not significantly align, with GPT-4 Turbo producing a higher number of “unanswerable” responses compared to humans. Notably, humans effectively leverage visual and auditory modalities to arrive at correct answers, resulting in many cases where humans answered correctly while GPT-4 Turbo marked the question as unanswerable. This discrepancy can be attributed to the limited input provided to GPT-4 Turbo, which consisted of only five frames sampled from approximately one minute of video, and the loss of critical information when the images are converted into simplified captions. As a result, GPT-4 Turbo often lacks the necessary information to form a response. Indeed, the majority of GPT-4 Turbo’s “unanswerable” judgments were due to “insufficient information”.

Qualitative Results for GPT-4 Turbo

**Figure 3:**
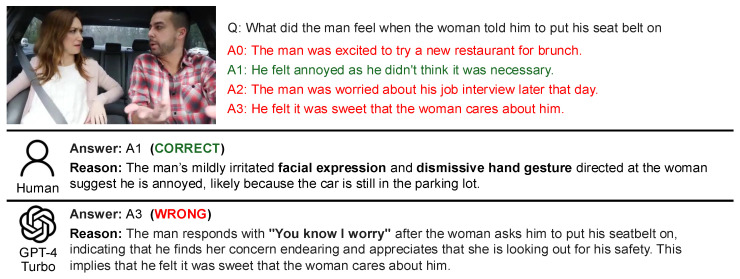
Example: Cases where humans answered correctly while GPT-4 Turbo provided a wrong answer.

**Figure 4:**
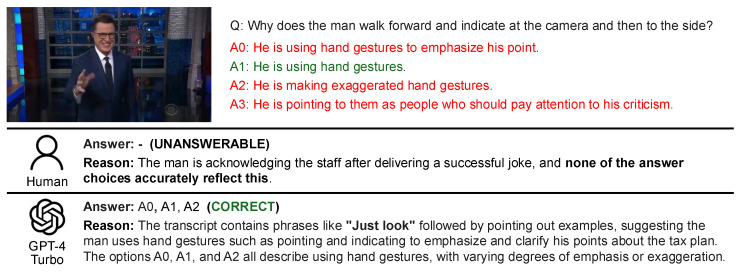
Example: Cases where humans marked the question as unanswerable while GPT-4 Turbo answered correctly.

An analysis was conducted on the response patterns arising from the combinations of Human: correct, wrong, unanswerable and GPT-4 Turbo: correct, wrong, unanswerable. The analysis comprised two components: (1) a manual review of five samples for each pattern, and (2) a trend analysis using GPT-4 Turbo on the complete set of samples. For the manual analysis, five samples were randomly selected for each pattern from the video–question pairs. Each sample was examined to review the question, the options, the human responses with their reasoning, and GPT responses with their reasoning. For the GPT-4 Turbo-driven trend analysis, the system was provided with the questions, options, human responses with their reasoning, and GPT responses with their reasoning for the video–question pairs in each pattern. The system was then instructed via a prompt to output findings under three categories: (1) commonalities between humans and the LLM, (2) differences between humans and the LLM, and (3) the characteristics of Social-IQ 2.0 QA. Subsequently, the outputs for each pattern were manually reviewed and synthesized into a comprehensive summary. The actual data and responses used in the analysis are presented in Figure 3 and Figure 4.

As shown in Figure 3, humans often rely on visual information to justify their answers, whereas GPT-4 Turbo primarily depends on dialogue information. This reliance leads GPT-4 Turbo to make literal interpretations of the text, resulting in frequent errors when visual cues are absent. In particular, Figure 3 illustrates a case where GPT-4 Turbo incorrectly attributed a statement made by a female speaker to a male speaker. This highlights the significant impact of lacking audio modality and the absence of speaker-related information in the dialogue data provided to GPT-4 Turbo.

It is considered that multimodal learning is an effective strategy for enhancing multimodal inference capabilities in large language models (LLMs). This is supported by the results in Table 1, which show that GPT-4o, despite having the same captions and transcript as inputs, achieved higher accuracy than GPT-4 Turbo due to its engagement in multimodal learning. Additionally, considering the difficulty and inefficiency of inputting extensive information from long-duration videos for high-accuracy inference, an approach like VideoAgent [53], which uses sparse input information to narrow down the temporal reasoning scope and retrieves only the necessary segments, would also be effective.

As shown in Figure 4, humans are sometimes able to identify the inappropriateness of options based on an understanding of the overall flow of the video. This tendency was not observed in GPT-4 Turbo. Instead, GPT-4 Turbo tends to base its answers on specific statements within the dialogue, selecting responses that do not contradict the identified evidence. This highlights GPT-4 Turbo’s behavior of answering only when clear evidence is found in the dialogue, with minimal subjectivity or inference. It predominantly interprets dialogue literally, relying strictly on the explicit information provided.

Based on the results of these analyses, the key characteristics of responses from GPT-4 Turbo and humans can be summarized as follows.

Characteristics of Human Responses
Positive characteristics

- Utilization of non-verbal information —Unlike LLMs, humans retain all information related to the video and excel in reasoning that integrates multiple modalities.
- Understanding of scene context—Humans can judge based on the norms of interpersonal interactions in various scenes.
- Understanding the temporal dynamics of emotions and relationships—Compared to LLMs, humans perform high-level temporal reasoning, understanding changes in emotions, relationships, and situations throughout the video, not just temporary feelings.
- Consideration of cultural background—Humans tend to consider cultural backgrounds even without specific instructions, taking into account various possibilities.
- Consideration of the overall narrative or scenario—Humans have the ability to imagine parts not included in the video, thus considering the overall narrative, including the premises and goals of the group involved in the scenes before and after the video.
- Marking questions as unanswerable—Humans can comprehensively assess the information related to the video and assert the inappropriateness of questions or options with clear justification.

Negative characteristics

- Prioritization of general interpersonal tendencies—When interpreting deep psychological states inferred from facial expressions or speech, humans may prioritize the norms of interpersonal relationships over specific details from the video.
- Risk of subjective interpretations—Humans may create unique interpretations or imaginations about the scenario, which can lead to overly subjective answers.

Characteristics of GPT-4 Turbo Responses
Positive characteristics

- Cautious approach when information is insufficient—GPT-4 Turbo refrains from answering when there is not enough evidence in the provided information.
- Literal interpretation of dialogue information—It interprets dialogue information literally, thus avoiding overly subjective answers.
- Detailed explanations and an elimination-based approach—Compared to humans, it provides more detailed justifications at the level of specific utterances, frequently referencing inappropriate answer options.
- Estimation of the conversation tone, scene atmosphere, and central figures—It can often sufficiently infer the tone of the conversation (e.g., positive or negative), the atmosphere of the scene, and central figures from dialogue information alone.

Negative characteristics

- Lack of visual context and audio cues—GPT-4 Turbo has a strong dependence on dialogue information, making it difficult to answer questions requiring visual context or audio cues.
- Deficiencies in temporal reasoning—LLMs still face challenges in temporal reasoning for long-duration video understanding, occasionally basing answers on parts not relevant to the question’s timing.
- Challenges in high-level psychological understanding—Due to limitations in utilizing multiple modalities and reasoning capabilities, GPT-4 Turbo often interprets statements literally and does not achieve a high-level understanding of social interactions and emotions, such as pleasantries.
- Lack of flexibility—If the dataset creation results in identical options appearing, GPT-4 Turbo might select only one despite the possibility of choosing multiple, thus overlooking others.

Quantitative

**Figure 5:**
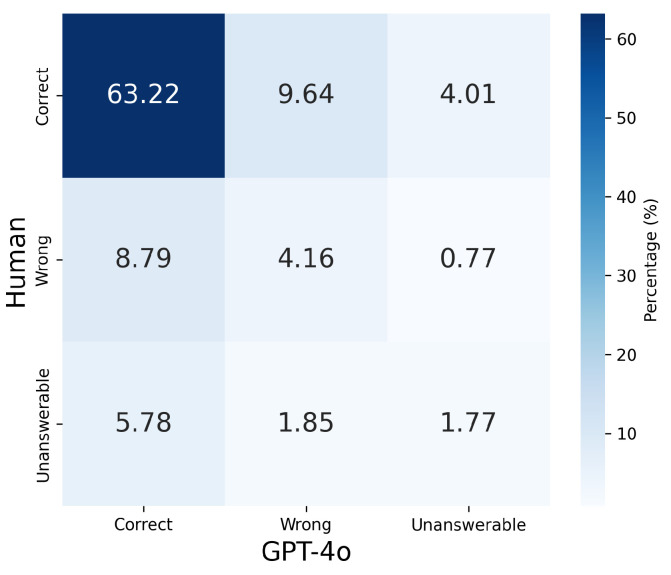
Visualization of human and GPT-4o performance. The chart illustrates the proportion of correct, wrong, and unanswerable responses in the overall dataset, with darker shades indicating higher proportions.

Results for GPT-4o GPT-4o was also evaluated using the same approach as GPT-4 Turbo, conducting a quantitative assessment of response patterns based on correctness. The analysis examined how GPT-4o performed across different response types, including correct, wrong, and unanswerable categories, to compare its tendencies with those of GPT-4 Turbo and humans. The results are presented in Table 3 and Figure 5.

From Table 3 and Figure 5, it can be observed that GPT-4o exhibits response tendencies that are closer to those of humans compared to GPT-4 Turbo. There are more instances where both GPT-4o and humans answered correctly or marked the question as unanswerable. Additionally, compared to GPT-4 Turbo, GPT-4o demonstrated fewer unanswerable responses and more correct answers for samples where humans either answered wrongly or marked the question as unanswerable. This improvement can likely be attributed to the differences in the amount of information captured by the images and captions, as well as the impact of multimodal pretraining, which enhances GPT-4o’s ability to integrate and effectively utilize information from multiple modalities.

Qualitative Results for GPT-4o

**Figure 6:**
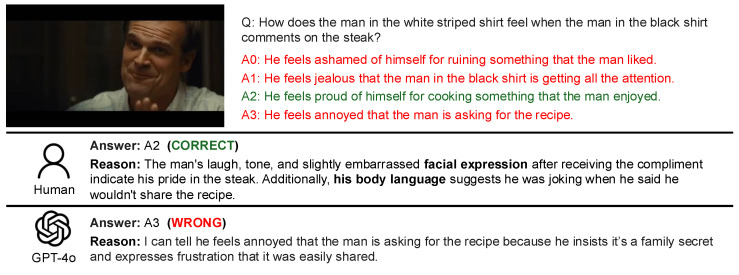
Example: Cases where humans answered correctly while GPT-4o provided a wrong answer.

**Figure 7:**
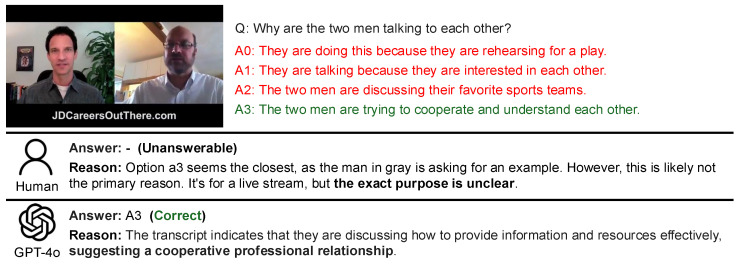
Example: Cases where humans marked the question as unanswerable while GPT-4o answered correctly.

The outputs of GPT-4o were qualitatively analyzed using both human judgments and GPT-4 Turbo-based evaluations, similar to the approach used for GPT-4 Turbo. As in previous sections, examples of the samples used for analysis are presented in Figure 6 and Figure 7. These examples illustrate specific cases that highlight the response tendencies and characteristics of GPT-4o.

As shown in Figure 6, GPT-4o can misinterpret questions when relevant visual information for the specific timing of the query is unavailable. In this example, as indicated by the human rationale, facial expressions or actions are key to determining the correct answer. Like GPT-4 Turbo, GPT-4o struggles with such samples where the visual context plays a critical role in answering accurately.

As shown in Figure 7, humans tend to avoid answering when they do not understand the premise or purpose of a scene or situation in the video. While this “unanswerable” tendency is similar to that of LLMs in cases of insufficient information, there is a key difference: LLMs typically mark a question as unanswerable when critical information within the video is missing. In contrast, humans often consider broader contextual or background information, even beyond what is explicitly presented in the video, before deciding that a question cannot be answered. This distinction highlights the differing approaches to reasoning between humans and LLMs.

The characteristics of GPT-4o revealed through the analysis are presented below.

Characteristics of GPT-4o Responses
Positive Characteristics

- Objective answer selection based on dialogue content—GPT-4o primarily infers correct answers from dialogue information, thereby avoiding unnecessary speculation and selecting answers based on statements.
- Estimation of tone and central figures from dialogue content—Similar to GPT-4 Turbo, GPT-4o can infer the tone of the conversation and identify the central figures from dialogue content alone, even without audio information.

Negative Characteristics

- Dominance of weaker expressions in option selection—Whereas humans might choose strong or specific words, GPT-4o tends to select objective and restrained expressions. This tendency, shared with GPT-4 Turbo, could be due to LLMs being configured to avoid overstatements and outputs that contradict compliance.
- Inability to point out issues without explicit instructions in the prompt—Similar to GPT-4 Turbo, LLMs do not perform reasoning beyond the instructions given; without clear directions, they cannot point out the inappropriateness of questions or answer options.

In addition, we discuss the insights obtained from the qualitative analysis of the QA properties of Social-IQ 2.0. In this study, we summarize the challenges of Social-IQ 2.0, particularly as insights for creating new benchmarks.

Characteristics of QA
Areas with Potential for Improvement

- Formulating questions based on a superficial interpretation of dialogue—Questions and options often fail to consider the scene context, such as being part of a comedy show or the relationship among workplace colleagues, representing only a general recognition of emotions without considering high-context information.
- Questions involving ambiguous emotions or concepts—Some questions address ambiguous emotions or concepts, making it difficult to distinguish between correct and incorrect options.
- Uncertainty in referencing scenes—Even for videos involving changes in emotions, there are questions that ask about emotions without specifying the exact moment, leading to unclear scene references.
- Extreme expressions or overly detailed options tend to be incorrect—Options containing extreme expressions or excessive detail are likely to be incorrect, with correct answers often identified through pattern learning rather than reasoning.
- Errors in question creation—Issues in QA creation include options that do not match the transcript, options that are difficult to semantically distinguish, or those that are identical in wording, leading to errors.

#### 3.3.2. Examination by Question Types

In this section, to gain insights for creating a new dataset, an analysis was conducted on the differences in the number of samples and accuracy across various question types. Questions were categorized into three types using GPT-4: (1) Timestamp, which includes questions about specific moments or scenes in the video; (2) Emotion, which involves questions about the emotions of a specific person and the context behind those emotions; and (3) Cause, which covers questions about the reasons behind particular statements or actions, focusing on causal relationships.

**Figure 8:**
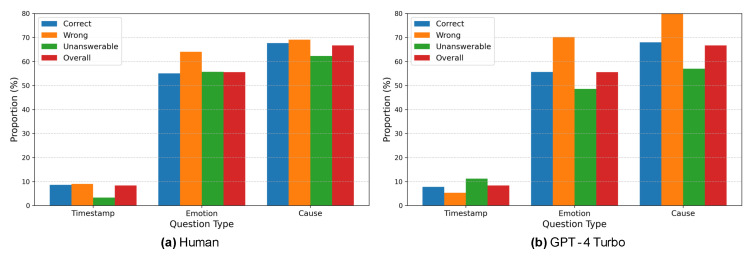
The percentages of human results (**a**) and GPT-4 Turbo results (**b**) categorized by question type. The blue bar graphs represent the proportion of correct samples for each question type—Timestamp, Emotion, and Cause, from left to right. Orange bars indicate the proportions of wrong responses, and green bars represent unanswerable responses, displayed similarly. The red bar graphs show the proportion of each question type relative to all samples. Deviations from the values in the red bars indicate particularly high or low accuracy.

**Figure 9:**
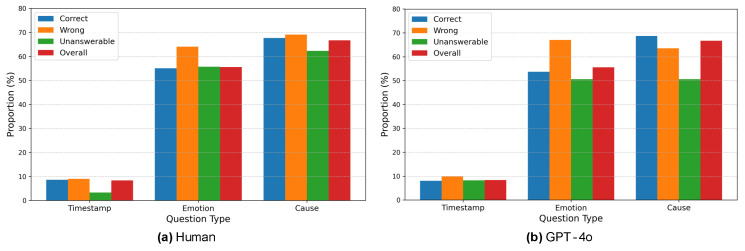
The percentages of human results (**a**) and GPT-4 Turbo results (**b**) categorized by question type. The blue bar graphs represent the proportion of correct samples for each question type, Timestamp, Emotion, and Cause, from left to right. Orange bars indicate the proportions of wrong responses, and green bars represent unanswerable responses, displayed similarly. The red bar graphs show the proportion of each question type relative to all samples. Deviations from the values in the red bars indicate particularly high or low accuracy.

The analysis examined the distribution of these question types in the dataset and the accuracy for each type. The results of the comparison between GPT-4 and humans are presented in Figure 8, while the comparison between GPT-4o and humans is shown in Figure 9. The accuracy for each question type is shown in Table 4.

From Figure 8, it is evident that humans have remarkably fewer unanswerable samples in questions involving timestamps. As seen from the short average reference time of videos in Table 5, questions with timestamps often have evidence appearing immediately before or after the specified point, making it easier to utilize precise visual or auditory information. Additionally, the inclusion of a timestamp eliminates ambiguity regarding the specific moment the question refers to. However, questions involving timestamps account for only about 8% of the total, meaning that in most cases, one must analyze the entire video to predict the relevant scene’s timing. This contributes to the challenging nature of the Social-IQ 2.0 benchmark. Furthermore, in questions about emotions or causes, the number of wrong answers exceeds the number of unanswerable cases. Combined with the qualitative evaluation of human responses in Section 3.3, this suggests that humans tend to incorporate external knowledge and thus are more prone to reflecting subjective interpretations.

As shown in (b), GPT-4 Turbo demonstrates a tendency to avoid wrong answers and produce “unanswerable” outputs in cases involving timestamps. This indicates that GPT-4 Turbo can accurately judge when information is insufficient, provided the relevant moment is clearly identified. Conversely, in questions without timestamps, GPT-4 Turbo is at risk of using irrelevant scenes as the basis for its answers. Additionally, the high number of wrong answers in questions about emotions or causes may stem from GPT-4 Turbo’s literal interpretation of dialogue, its inability to infer underlying intentions from facial expressions, and its failure to correctly associate characters’ appearances with their statements.

From Figure 9, it can be observed that GPT-4 Turbo has fewer unanswerable cases compared to humans and GPT-4 Turbo. This is evident from the short average reference time of videos in Table 5, as questions with timestamps often have supporting evidence appearing immediately before or after the specified point, making it easier to utilize precise visual or auditory information. Additionally, GPT-4o tends to make more wrong answers in timestamp-based questions but performs better in questions about causes, showing an opposite trend to GPT-4 Turbo. This conclusion, when combined with the qualitative results, indicates that the provision of a timestamp facilitates the identification of necessary information and GPT-4o attempts to answer even when there is insufficient information.

For questions about causes, GPT-4o has shown higher accuracy compared to humans. This can be considered, based on qualitative results, to reflect GPT-4o’s characteristic of making literal interpretations of dialogue and being less susceptible to subjective biases than humans.

From Table 4, it can be seen that GPT-4 Turbo’s accuracy on timestamp-based questions is 15–20% lower than that of humans and GPT-4o. This is likely because the specified timestamp allows GPT-4 Turbo to clearly determine whether sufficient information is available. Conversely, when GPT-4 Turbo does provide an answer, its accuracy is exceptionally high at 88.89%. This indicates a cautious approach, where GPT-4 Turbo only answers when it determines that sufficient evidence is present, demonstrating a trend distinct from GPT-4o. Furthermore, the high accuracy when GPT-4 Turbo does answer is partly due to its responses being less influenced by subjective biases.

For emotion-related questions, GPT-4o performs at a similar accuracy level to humans, despite having access to limited visual information. This is likely because humans tend to refrain from answering based on external context, such as general tendencies in social interactions (e.g., adults behaving politely), which can lead to a more conservative approach.

#### 3.3.3. Analysis of Options Discriminability

**Figure 10:**
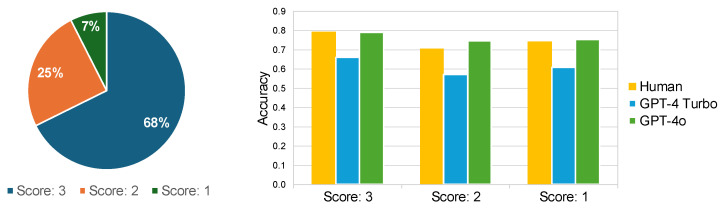
Accuracy by the discriminability of options. The scoring is defined as follows: a score of 3 indicates that all four options are semantically distinguishable; a score of 2 denotes that two or more options are not semantically distinguishable; and a score of 1 signifies that two or more options are completely identical.

In this section, the relationship between QA quality and accuracy was examined by evaluating the distinguishability of options for each QA pair. These evaluations were conducted by GPT-4 Turbo using a three-point scale. Subsequently, the proportion of each score across all samples and the accuracy of humans and LLMs for each score were calculated. The results are presented in Figure 10.

As shown in Figure 10a, only 68% of the questions have semantically distinguishable options. This raises concerns about the reliability of accuracy assessments in the Social-IQ 2.0 benchmark. Furthermore, despite the setting of this study allowing for multiple output options, as seen from Figure 10b, there was a 5–10% decrease in accuracy in cases with scores of 2 or 1 compared to cases with a score of 3. These findings indicate the significant impact of option discriminability on results and the limitations of LLMs’ flexibility. Considering the inherent ambiguity and subjectivity in tasks related to social intelligence, it is appropriate to include not only the correct options but also the reasons in the benchmarks.

### 3.4. Ablation Study

In this section, an ablation study was conducted using the Social-IQ 2.0 dataset to gain insights into appropriate configurations for applying LLMs to VideoQA tasks. The specific factors examined included the types of input data, the number of frames, sampling strategies, variations in captioning models, and differences in caption granularity.

#### 3.4.1. Input Data Type

First, the impact of input data types on QA accuracy was validated by applying GPT-4 Turbo and GPT-4o to videos contained in the Social-IQ 2.0 Sub dataset. The experimental setup was almost identical to that described in Section 3.3, using data from five frames evenly spaced out, excluding the first and last 20 frames, as the regular visual information. However, the “mages (large)” experiment involved validation using only a large volume of visual data. Due to the limitations on the amount of input data, it was not possible to input all frames of the video, so instead, 141 frames were used, excluding the first and last 20 frames. As points of comparison, the results of humans answering solely from dialogue information or from video are also documented.

From the comparison between humans and LLMs in Table 6, it is evident that humans have significantly lower accuracy when answering only from the transcript. This suggests that, unlike LLMs, which heavily rely on the dialogue modality, humans tend to integrate multiple modalities in their reasoning. Furthermore, GPT-4o has surpassed the QA accuracy of humans who answered from video, indicating that in the Social-IQ 2.0 benchmark, it is becoming difficult to distinguish between the social intelligence of LLMs and humans based on QA accuracy alone. However, this study has enabled evaluation methods beyond QA accuracy, revealing new differences between GPT-4o and LLMs, such as the number of unanswerable samples. This is thought to reflect the differences discussed in Section 3.3, regarding whether GPT-4o and humans can point out the inappropriateness of questions and answer choices with clear justification.

In addition, it can be observed that GPT-4 Turbo’s accuracy decreases with the addition of visual information, whereas GPT-4o’s accuracy increases. This indicates that GPT-4o, which engages in multimodal learning, can effectively combine visual information with textual information for reasoning. The difference in the amount of information provided by captions and images also contributes to these results. It is considered that using simple captions of about ten words might lead to overlapping information in frames, which in turn could act as noise. Moreover, GPT-4 Turbo is significantly affected by input data in terms of accuracy, while GPT-4o is relatively less affected by the input data. This aligns with observations made in Section 3.3, where GPT-4o tends to attempt answers even with insufficient information, whereas GPT-4 Turbo tends to avoid answering if it cannot find clear evidence from the input data. When comparing the input of 5 images versus 141 images in GPT-4o, it is observed that the accuracy decreases with 141 images. This is likely due to the inclusion of redundant information, which results in the image-related information becoming noise.

#### 3.4.2. Sampling Strategies and Frame Selection

This section examines the accuracy achieved by different combinations of frame numbers and sampling strategies. GPT-4 Turbo was used as the LLM, and BLIP-2 was employed as the captioning model. The sampling strategies are defined as follows:

- Central Interval: Frames are evenly sampled from the middle portion of the video, excluding the beginning and end (e.g., sampling from frames 20–160 out of approximately 180 frames).
- Uniform: Frames are evenly sampled across the entire video.
- Random: Frames are randomly sampled from the entire video.
- Random (sorted): Frames are randomly sampled from the entire video and then sorted in index order.

The results are presented in Table 7.

When GPT-4 Turbo was used as the LLM and BLIP-2 as the captioning model, the accuracy was higher when the number of frames was zero or one. However, as the number of frames increased, accuracy fluctuated rather than consistently improved, indicating that increasing the frame count was not particularly beneficial. This can be attributed to the captions generated by BLIP-2, which tend to be concise and lack substantial information. Including more frames often failed to provide additional information and, in some cases, introduced noise instead.

Regarding sampling strategies, the best average accuracy was observed when frames were randomly extracted and then sorted in index order. This suggests that the variation introduced by unevenly spaced frames might lead to more diverse and informative captions compared to uniformly spaced sampling. The second-best performance was achieved with the “Central Interval” strategy, where frames were evenly sampled, excluding the beginning and end of the video. This is likely because the start and end of a video often contain transitional scenes with limited information, making them less useful for generating captions.

Next, the relationship between the number of frames and accuracy was investigated for each LLM. The LLMs used were GPT-4 Turbo and GPT-4o, with GPT-4o evaluated in two settings: using captions and using images. For the image-based evaluation, five frames were evenly sampled from the video, excluding the beginning and end. For the caption-based evaluation, BLIP-2 was applied to generate captions for these five frames. The results are shown in Table 8.

For GPT-4 Turbo, increasing the number of frames did not lead to a significant improvement in accuracy. In contrast, for GPT-4o, although the highest accuracy was observed when the number of frames was zero, accuracy improved as the number of frames increased. This finding highlights GPT-4o’s capability to integrate visual and textual information effectively, reflecting its strength in multimodal reasoning. Additionally, the results indicate that when the number of frames exceeds five, GPT-4o achieves higher accuracy when provided with images rather than captions. This can be attributed to the greater richness of information contained in images compared to captions and GPT-4o’s ability to appropriately link textual and visual information for improved inference.

As shown in Table 8, GPT-4o exhibited a tendency for accuracy to improve with an increasing number of frames. To further investigate this trend, the accuracy was evaluated with a larger number of frames, using the same “Central Interval” sampling strategy. The results are presented in Table 9.

From the results in Table 9, the highest accuracy was achieved when using 141 frames (utilizing all frames excluding the first and last). However, this accuracy is only 0.01 points higher than when using 15 frames. Moreover, the execution time for 141 frames was shorter than that for 21 frames, suggesting that not all images are being thoroughly analyzed. This implies that, even with a large number of frames, only a subset may actually be utilized for analysis. Therefore, it would be reasonable to limit the number of frames to a moderate level to balance performance and computational efficiency.

#### 3.4.3. Caption Models and Variations in Caption Granularity

In this section, to clarify the impact of caption content on accuracy, experiments were conducted on caption models and caption granularity. Five frames were used for the evaluation, with the “Central Interval” sampling strategy applied. Two caption models, BLIP-2 [52] and Instruct-BLIP [54], were used in this analysis. The types of captions were controlled through prompts, resulting in two variations:

- Short Captions: Approximately 10 words (e.g., “a group of men sitting at a dinner table”).
- Detailed Captions: Approximately 15–30 words (e.g., “a woman and a man are sitting at a table in front of a cityscape with the words ’socially connected’ written on it. The woman is talking to the man, who is wearing a suit”).

The results are summarized in Table 10.

From Table 10, it was observed that providing more detailed captions did not improve accuracy. This aligns with earlier findings that increasing the number of frames does not necessarily enhance accuracy. These results may reflect limitations in GPT-4 Turbo’s reasoning capabilities or the possibility that captions from irrelevant frames introduce noise. This suggests that the relevance of the captions to the questions is more important than the detail of the captions.

## 4. Discussion

In this study, we applied large language models (LLMs) such as LLaMA and GPT to the Social-IQ 2.0 dataset in a zero-shot setting. The results demonstrated that these models achieved accuracy comparable to state-of-the-art methods without requiring dataset-specific training, thereby highlighting their robustness and adaptability across diverse scenarios. Leveraging the capability of LLMs to generate customizable outputs, we conducted a detailed analysis using supplementary annotation data, uncovering differences in rationales and response patterns between humans and LLMs that surpass traditional QA accuracy metrics. These findings provide new insights that were previously unattainable with conventional Social-IQ benchmarks and end-to-end models, shedding light on the strengths and challenges of LLMs in social intelligence tasks.

Humans and LLMs exhibit distinct response patterns shaped by their inherent characteristics. Humans often consider the overall context of a video, including general tendencies in interpersonal relationships, when responding. They tend to approach ambiguous questions or scenarios with unclear premises cautiously, often refraining from answering in such cases. Additionally, humans sometimes overthink scenarios not included in the video or excessively incorporate external knowledge, leading to subjective responses. In such cases, this can often result in errors.

GPT-4 Turbo focuses on extracting clear reasoning from dialogue information. It consistently attempts to answer when it identifies explicit evidence in the input but refrains from doing so when such evidence is lacking, particularly in questions that rely on inference or non-verbal cues, such as tone or expressions. While GPT-4 Turbo can interpret the nuances and context of dialogue directly, it struggles with questions that require advanced reasoning or indirect implications.

GPT-4o has demonstrated a stronger ability to infer from limited information compared to GPT-4 Turbo, resulting in fewer cases of being unable to answer. GPT-4o performs particularly well in samples where humans provide correct answers and often delivers accurate responses to questions that humans cannot justify. However, compared to humans, GPT-4o lacks the ability to critique inappropriate questions and answer choices.

Ablation studies have revealed that visual information often introduces noise in GPT-4 Turbo and that processing a large number of frames (e.g., more than 15) is not efficient in GPT-4o either. Additionally, experiments on the granularity of captions emphasized that providing detailed captions does not enhance the accuracy of GPT-4 Turbo, highlighting the importance of capturing scene-relevant information.

In summary, this study suggests the high potential of LLMs in advancing video-based reasoning and social intelligence applications through their adaptability to various scenarios and capability to perform detailed analyses with free-form outputs, derived from the strong zero-shot performance of LLMs. Models like GPT-4 Turbo and GPT-4o possess excellent interpersonal understanding capabilities; however, there remains room for improvement in effectively integrating multimodal information, critiquing question design, and performing integrative reasoning beyond the given data. Additionally, the Social-IQ 2.0 Sub itself has issues such as the “unanswerable” criteria being highly subjective and a lack of sufficient evaluation metrics, making it difficult to comprehensively and accurately assess the social intelligence capabilities of AI. It is considered necessary to incorporate additional annotations and introduce new evaluation metrics in the future.

Future research directions can broadly be divided into two categories: (1) proposing new methodologies and (2) devising new benchmarks and evaluation methods. However, it is imperative to focus on the latter due to the significant advancements in large language models (LLMs). This is because the significant advancements in LLMs have brought the QA accuracy of GPT-4o in Social-IQ 2.0 to a level comparable to that of humans, making it increasingly meaningless to simply aim for higher accuracy within the confines of Social-IQ 2.0. Considering the inherent subjectivity and ambiguity in social intelligence, creating QA tasks with definitive correct answers is nearly impossible. Therefore, it is crucial to develop benchmarks that include rationales, enabling learning systems to handle the ambiguity inherent in human relationships with human-like reasoning. Additionally, devising evaluation metrics for each component of social intelligence, such as the social appropriateness of emotions and behaviors, understanding of scene context, as well as developing methods to assess the consistency of responses and the similarity in reasoning approaches, could also become interesting topics of research.

Given the improvements in LLMs’ multimodal reasoning capabilities and their ability to reason over complex, long-duration videos, a modular approach that allows for the generation of intermediate outputs is more suitable than traditional end-to-end models, which struggle with detailed analysis. Without datasets that are temporally and spatially grounded, it is impossible to evaluate these intermediate outputs. Consequently, there is an urgent need to develop benchmarks with denser annotations compared to traditional video benchmarks.

Should LLMs achieve a high level of multimodal interpersonal communication through such processes, several applications could be realized. These include natural communication with caregivers, customer service that conducts appropriate dialogues through monitoring user responses, coaching in hospitality or acting, and emotional coaching for patients with Autism Spectrum Disorder (ASD) or young children.

## 5. Conclusions

In this study, we applied LLMs to the Social-IQ 2.0 dataset in a zero-shot setting and demonstrated that these models achieve accuracy comparable to state-of-the-art methods without requiring dataset-specific training. This highlights their robustness and adaptability across diverse scenarios. By leveraging supplementary annotation data, we conducted detailed comparisons between humans and LLMs, revealing differences in response rationales and patterns that go beyond conventional QA performance metrics. Additionally, through a comprehensive analysis of input data, frame count, sampling strategies, and caption types, we provided practical guidelines for effectively integrating LLMs into video-based AI tasks. These findings underscore the potential of LLMs in advancing video-based reasoning and social intelligence applications, laying the groundwork for broader adoption and further research in this domain.

## Figures and Tables

**Table 1:** Comparison of QA accuracy between existing methods and LLMs. The term Presence of Training refers to whether or not training was conducted using the Social-IQ 2.0 dataset. The term Input Data refers to data entered in addition to the question and options.

Model	Presence of Training	Input Data	Accuracy (%)
Just Ask Plus	without	images + transcript	53.4
DeSIQ	with	video	62.28
MMTC-ESC	with	audio + video	75.94
Llama-3-8B-Instruct	without	captions + transcript	52.42
gpt-3.5-turbo	without	captions + transcript	56.75
gpt-4	without	captions + transcript	64.56
gpt-4-turbo	without	captions + transcript	70.26
gpt-4o-mini	without	images + transcript	66.91
gpt-4o	without	captions + transcript	73.23
gpt-4o	without	images + transcript	75.71

**Table 2:** Comparison of human and GPT-4 Turbo performance. “# Correct” represents the number of correctly answered questions, “# Wrong” represents the number of incorrectly answered questions, “# Unanswerable” represents the number of unanswerable questions, and “# Total” represents the total number of questions.

	LLM	# Correct	# Wrong	# Unanswerable	# Total
Human					
# Correct		682	91	224	997
# Wrong		85	42	51	178
# Unanswerable		58	18	46	122
# Total		825	151	321	1297

**Table 3:** Comparison of human and GPT-4o performance. “# Correct” represents the number of correctly answered questions, “# Wrong” represents the number of incorrectly answered questions, “# Unanswerable” represents the number of unanswerable questions, and “# Total” represents the total number of questions.

	LLM	# Correct	# Wrong	# Unanswerable	# Total
Human					
# Correct		820	125	52	997
# Wrong		114	54	10	178
# Unanswerable		75	24	23	122
# Total		1009	203	85	1297

**Table 4:** Accuracy by question types; “when answered” refers to the accuracy excluding samples that are unanswerable.

Metric	Timestamp (%)	Emotion (%)	Cause (%)
Human Accuracy	79.63	76.14	78.03
GPT-4 Turbo Accuracy	59.26	63.66	64.86
GPT-4o Accuracy	75.00	75.17	80.12
Human Accuracy (when answered)	82.69	84.07	85.55
GPT-4 Turbo Accuracy (when answered)	88.89	81.24	82.26
GPT-4o Accuracy (when answered)	80.20	79.94	84.31

**Table 5:** Humans’ average video reference time by question types.

Average Video Reference Time	Timestamp (s)	Emotion (s)	Cause (s)
From video	7.40	27.51	22.79
From transcript	11.60	23.30	20.30

**Table 6:** Validation results by changing input data types. “QA” refers to the question and four answer options; “trans.” is an abbreviation for “transcript”; “captions/images” correspond to data from five frames evenly spaced out, excluding the first and last 20 frames; “images (large)” refers to data from 141 frames, also evenly spaced out but excluding the first and last 20 frames. “# Correct” represents the number of correctly answered questions, “# Wrong” represents the number of incorrectly answered questions, and “# Unanswerable” represents the number of unanswerable questions.

Model	Input Data	# Correct	# Wrong	# Unanswerable	Accuracy (%)
GPT-4 Turbo	QA	538	255	504	41.48
GPT-4o	QA	813	383	101	62.68
GPT-4 Turbo	QA + trans.	836	162	299	64.46
GPT-4o	QA + trans.	994	162	141	76.64
Human	QA + trans.	488	146	663	37.63
GPT-4 Turbo	QA + captions	76	18	1203	5.86
GPT-4o	QA + images	770	301	226	59.37
GPT-4o	QA + images (large)	747	294	256	57.59
GPT-4 Turbo	QA + trans. + captions	825	151	321	63.61
GPT-4o	QA + trans. + images	1009	216	71	77.79
Human	video	997	178	122	76.87

**Table 7:** Validation of frame numbers and sampling strategy. “# Frames” represents the number of input frames. In the table, the cell with the highest accuracy for each sampling strategy is highlighted with bold borders, and the sampling strategy with the highest accuracy for each frame number is underlined.

LLM	Caption Model	# Frames	Central Interval	Uniform	Random	Random (Sorted)
GPT-4 Turbo	BLIP-2	0	**64.46%**	**64.46%**	64.46%	64.46%
		1	63.15%	63.15%	**64.92%**	**64.92%**
		3	64.30%	63.53%	62.84%	63.84%
		5	63.61%	63.30%	63.38%	63.69%
		8	63.99%	62.60%	63.30%	64.76%

**Table 8:** Validation of frame numbers and LLM types. “# Frames” represents the number of input frames.

# Frames	GPT-4 Turbo	GPT-4o (Captions)	GPT-4o (Images)
0	64.46%	76.64%	76.64%
1	63.15%	74.48%	46.88%
3	64.30%	74.10%	73.79%
5	63.61%	74.02%	77.79%
8	63.99%	75.94%	77.87%

**Table 9:** Verification of large-frame input handling in GPT-4o. “# Frames” represents the number of input frames.

# Frames	Accuracy (%)	Execution Time (s)
11	78.64	6819
15	79.25	8340
21	78.87	9589
141	79.26	9410

**Table 10:** Comparison of LLMs by caption types.

LLM	Caption Model	Short Caption	Detailed Caption
GPT-4 Turbo	BLIP-2	63.61%	61.22%
	Instruct-BLIP	63.30%	61.06%

## Data Availability

Data are contained within the article.

## Abbreviations

The following abbreviations are used in this manuscript:

AI	artificial intelligence
QA	Question Answering
LLM(s)	Large Language Model(s)
VCR	Visual Commonsense Reasoning
MCQA	Multimodal Co-Attention Based Network for Question Answering
MMTC-ESC	Multi-Modal Temporal Correlated Network with Emotional Social Cues
ASD	Autism Spectrum Disorder
